# Multi-Factor Prediction of Cumulative Deposited Height in CMT Wire Arc Additive Manufacturing Using an Improved Temporal Convolutional Network

**DOI:** 10.3390/ma19194169

**Published:** 2026-09-29

**Authors:** Yuwen Wang, Qikuan Zhao, Haocheng Wu, Longjian Zhou, Hao Deng, Yu Fan, Xue Li, Jie Xu, Zheng Chen, Lin Wang

**Affiliations:** 1 School of Materials Science and Physics, China University of Mining and Technology, Xuzhou 221116, China; yuwen.wang@cumt.edu.cn (Y.W.); 18852130780@163.com (H.D.); fanyu@cumt.edu.cn (Y.F.); chenzheng1218@163.com (Z.C.); 2 MOE Key Laboratory for Liquid-Solid Structure Evolution and Materials Processing, Institute of Materials Joining, Shandong University, Jinan 250061, China; 202114097@mail.sdu.edu.cn

**Keywords:** wire arc additive manufacturing, deposited height, multi-factor features, temporal convolutional network, feature-wise linear modulation, causal attention

## Abstract

Cold Metal Transfer wire arc additive manufacturing (CMT-WAAM) efficiently fabricates medium and large metallic components, yet deposited wall cumulative height varies spatially along the build direction and deposition path due to heat accumulation, inter-layer cooling, travel direction and inherited layer geometry. This study establishes a multi-factor prediction framework for single-bead multi-layer walls and evaluates CAFi-TCN, a temporal convolutional network enhanced with feature-wise linear modulation and causal attention. Height profiles were extracted from registered point clouds under 2–4 mininter-layerr cooling; the model uses deposition position, layer number, cooling time, travel direction, prior height increment and cumulative height to predict current-layer cumulative height. On the tenth-layer test set, CAFi-TCN achieved the lowest mean absolute error (MAE = 0.1836 mm) among the evaluated direct-height models, reducing MAE by 74.3%, 40.4% and 53.9% versus standard TCN, polynomial ridge regression and MLP, respectively. A Random Forest model trained on the height-increment target (RF-Δ) produced slightly higher MAE but lower RMSE and maximum absolute error, showing a trade-off between average-error control and extreme-error suppression. Additional no-PreDH, simple increment-baseline, path-block bootstrap and rolling-layer analyses show that prediction performance depends on both process-state variables and inherited geometry rather than simple copying of the previous layer. The results support bounded, layer-wise height forecasting for single-bead WAAM walls and provide a basis for pre-adjustment error identification.

## 1. Introduction

Wire arc additive manufacturing (WAAM) uses an electric arc as the heat source and wire as the feedstock. It offers high deposition efficiency, relatively open equipment integration and clear advantages for medium and large metallic parts [1,2]. Cold Metal Transfer (CMT) is a low-heat-input arc process that is widely used as a WAAM heat-source variant because it provides stable metal transfer and reduced thermal input. Metal additive manufacturing is nevertheless governed by coupled effects of heat input, material accumulation, toolpath planning and repeated thermal cycling [3,4]. For single-bead multi-layer thin-wall components, the cumulative deposited height affects inter-layer overlap, surface profile, torch stand-off distance, arc stability and the deposition state of the next layer. Predicting deposited height is therefore a practical prerequisite for geometric control, trajectory compensation and pre-adjustment of process parameters in WAAM.

Research on WAAM geometry control has developed along several lines, including process monitoring, thermo-mechanical analysis, path planning and closed-loop control. Reviews of WAAM monitoring, automation and artificial-intelligence applications show that geometric quality is shaped by both process stability and data acquisition capability [5,6,7]. Simulation-oriented studies further indicate that thermal history and deposition trajectory jointly affect the final build state [8,9]. Recent closed-loop and arc-voltage-sensing studies have also emphasized that vision, arc-state sensing and controller design must be considered together when improving WAAM geometric consistency [10,11]. These studies indicate that the geometry of a WAAM wall is not a purely local layer response, but a result of process conditions, path-dependent behaviour and inherited thermo-geometric states.

Bead geometry prediction and layer-wise process control provide a direct basis for height modelling. Closed-loop control studies have shown that geometric deviation can be fed back to subsequent layers, while layer-superposition modelling has described how single-bead geometry accumulates in multi-layer walls [12,13]. Machine-learning studies have used point-cloud or bead-geometry data to predict process parameters and bead dimensions in WAAM [14,15]. More recent geometry-prediction work has extended this idea to corner structures and three-dimensional wall deposition, highlighting the need to account for path shape, thermal history and deposition sequence [16,17]. These studies suggest that cumulative height prediction should include not only local deposition geometry, but also layer-wise states and the previous geometry of the wall.

Vision sensing and data-driven modelling have further improved the observability of metal additive processes. Molten-pool images have been used to infer weld reinforcement in arc additive manufacturing, and in situ optical monitoring has shown that visual signals can capture WAAM process variations [18,19]. More recently, attention-based temporal convolutional networks have been adopted for future deposition-height prediction in directed energy deposition, and physics-driven temporal convolutional models have been used for melt-pool width and layer height prediction [20,21]. Data-driven studies on contour consistency and sequential as-deposited geometry prediction further show that profile prediction is moving from isolated point estimation to sequence-aware modelling of layer geometry [22,23].

Recent WAAM monitoring studies have also extended the sensing basis from single optical images to point-cloud defect evaluation, unsupervised process-state monitoring and real-time defect detection [24,25,26]. These studies reinforce the need to connect measured geometry, process state and model outputs in a reproducible data pipeline. Sensor-integrated acquisition platforms and open-source WAAM systems further show that future height-control studies should report both manufacturing parameters and computational implementation details [27,28,29]. Reviews of in situ monitoring and machine-learning control reach the same conclusion: prediction accuracy must be interpreted together with sensor uncertainty, data structure and process scope [30,31].

Cumulative height prediction for a single-bead multi-layer WAAM component remains difficult for three reasons. First, the height at a given position depends on layer number, inter-layer cooling time (CT), travel direction and the geometry carried over from previous layers. Second, start and end regions do not share the same error pattern as the stable middle region; a single global metric can therefore hide local risks. Third, static nonlinear regressors and standard sequence models do not explicitly couple condition-dependent feature interpretation with historical geometric dependence and path-direction nonuniformity. For cumulative-height modelling, the measured path profile can be regarded as a spatial sequence rather than a conventional time series. Its order along the deposition path nevertheless follows the torch motion: each downstream position is formed after the upstream position and is affected by the preceding thermal and geometric state. This causal path-wise ordering makes temporal convolution suitable for cumulative height profiles. Dilated temporal convolutions support long-range ordered-sequence modelling [32]; LSTM provides a recurrent baseline for sequence learning [33], and attention mechanisms select relevant historical information [34]. Feature-wise conditioning has also been used to modulate intermediate neural-network features according to external variables [23,35], which motivates condition-modulated temporal modelling for deposited-height prediction.

Despite these advances, the cumulative height prediction problem in WAAM has not been fully resolved. Existing bead-geometry studies mainly focus on process-parameter-to-geometry mapping or single-layer bead dimensions, and they rarely describe how the measured wall profile is inherited by the next layer [14,15]. Recent sequence-learning studies show the value of ordered geometric information [20,21]. Profile-prediction studies further indicate that layer geometry should be treated as a spatially ordered output, but the roles of cooling time, deposition direction and previous-layer geometry are often not evaluated together in a single layer-wise prediction framework [22,23]. In addition, many data-driven studies report only global prediction accuracy, whereas start and end regions along a reciprocating WAAM path can produce localized deviations that are important for subsequent parameter adjustment [24,36,37]. These gaps make it necessary to build a model that combines process-state variables, historical geometry and path-wise sequence information, and to evaluate it with baseline comparison, regional error analysis and dependence checks on previous layer height.

This study builds a deposited-height dataset from a CMT-WAAM fabrication and layer-wise 3D scanning workflow, and evaluates CAFi-TCN for multi-factor height prediction. The contributions are as follows: (1) a feature system containing Loc (sampled position along the deposition path), N (current layer number), CT (inter-layer cooling time), Dir (travel direction), PreLH (height increment of the previous layer) and PreDH (cumulative deposited height of the previous layer) is defined to connect spatial position, process state and historical geometry; (2) CAFi-TCN combines FiLM-based condition modulation with a causal attention fusion block for path-wise historical information; (3) deep-learning, linear/polynomial, nearest-neighbour, tree-based and previous-height baselines are compared on the same tenth-layer test set, together with a no-PreDH audit, tree-model delta-target comparison and rolling target-layer validation; (4) conditional response and regional error analyses clarify how cooling time, end-region transients and historical geometry affect cumulative deposited-height prediction.

## 2. Materials and Methods

### 2.1. Input Features and Experimental System

**Figure 1:**
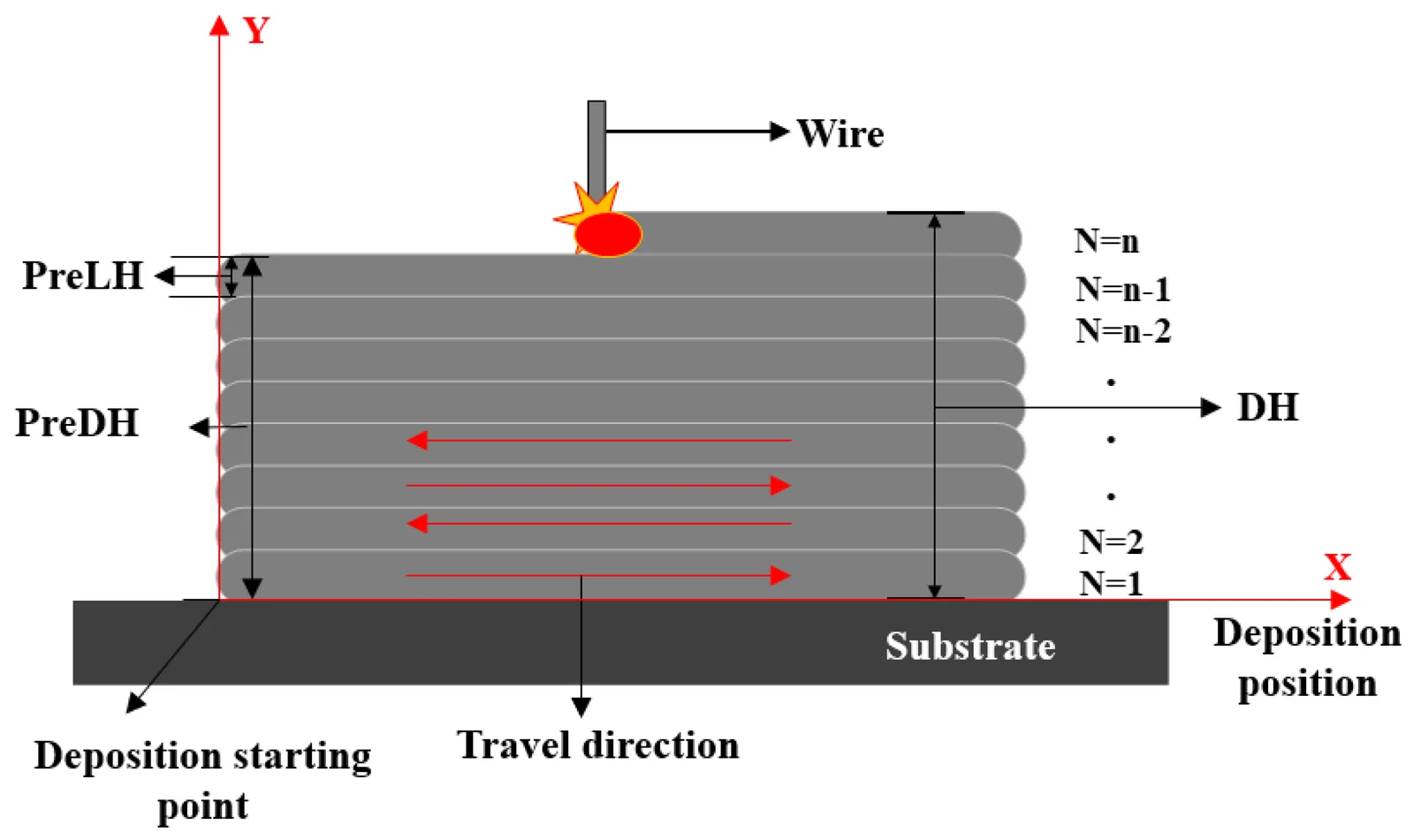
Multi-factor input features and the cumulative deposited-height prediction target.

As shown in Figure 1 and Table 1, the input variables and prediction target were defined on the ordered height sequence along the deposition path. This feature design keeps spatial, process-state and historical geometry information in a single prediction framework.

Model performance was evaluated using mean absolute error (MAE), root mean square error (RMSE), prediction accuracy (PA), coefficient of determination (R^2^) and maximum absolute error (MaxAE). MAE was used as the primary metric because the intended application is an average geometric-deviation assessment before path compensation. RMSE and MaxAE describe local large-deviation risk, while R^2^ provides an auxiliary measure of height-variation explanation. The metrics were calculated as: MAE = mean(|DH − DH_pred|), RMSE = sqrt(mean((DH − DH_pred)^2^)), PA = 1 − MAE/mean(|DH|), and R^2^ = 1 − sum((DH − DH_pred)^2^)/sum((DH − mean(DH))^2^).

In these expressions, DH denotes the measured cumulative deposited height and DH_pred denotes the predicted height. Because PA is scaled by the mean cumulative height, it is reported as an auxiliary index and is not used as the main basis for model ranking. In regions with small height variation, R^2^ should be interpreted together with MAE, RMSE and the path-wise error distribution. For tail-risk analysis, P50, P90 and P95 denote the 50th, 90th and 95th percentiles of the absolute prediction error, respectively; for example, P90 is the error threshold below which 90% of the test samples fall.

**Figure 2:**
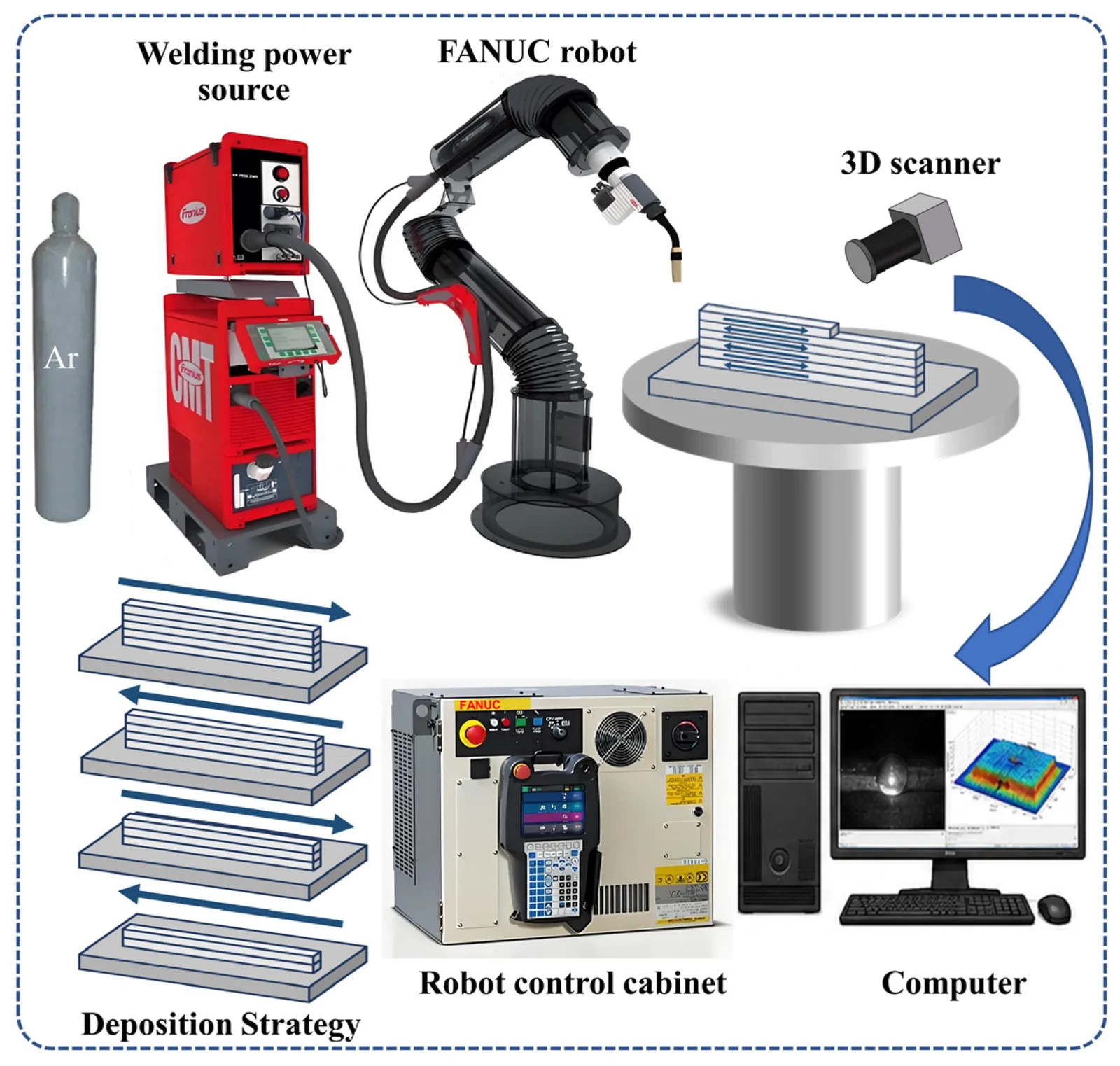
Schematic diagram of the component deposited-height prediction system, integrating the CMT-WAAM experimental setup with the data-driven prediction workflow.

The experiments were carried out using a unified CMT-WAAM fabrication and layer-wise 3D measurement workflow, as shown in Figure 2. The fabrication unit consisted of a FANUC welding robot (FANUC CORPORATION, Oshino-mura, Japan), a Fronius TPS 500i CMT power source (Fronius International GmbH, Pettenbach, Austria), a FANUC robot controller (FANUC CORPORATION, Oshino-mura, Japan), a wire feeder (Fronius International GmbH, Pettenbach, Austria) and a shielding-gas system. The measurement unit used a Revopoint POP 3 Plus 3D scanner (Revopoint 3D Technologies, Shenzhen, China) to acquire the wall geometry after each deposited layer, with the scanner-to-workpiece distance maintained at 20 cm; the measurement accuracy reported for this workflow was 0.04 mm. The registered 3D scan provided the cumulative-height labels used for model training and evaluation.

In the CMT wire arc additive manufacturing experiments, a Q235 low-carbon steel plate was used as the substrate, and a MG50-6 copper-coated carbon steel wire with a diameter of 1.2 mm was used as the filler wire; its chemical composition is listed in Table 2. The wire feed speed was set to 4.0 m/min, and the power source automatically matched the welding voltage and current to 14.6 V and 144 A, respectively. The shielding gas was 98% Ar + 2% CO_2_ at a flow rate of 18 L/min. The deposition travel speed was 30 cm/min. Each layer was deposited over a length of 100 mm, and 10 layers were deposited in total. The contact-tip-to-work distance (CTWD) was set to 15 mm and maintained constant throughout the deposition process.

The fabrication and 3D measurement steps were performed within the same layer-wise workflow. The CMT system deposited the wall layer by layer, and the 3D scanner provided the off-line geometric ground truth after each layer. This workflow links the process variables and the deposited-height labels at the layer level.

A reciprocating deposition strategy was adopted, so that adjacent layers were deposited in opposite travel directions. This strategy reduces the end-region asymmetry associated with one-way deposition, but it also means that the start region of one layer may spatially correspond to the end region of the next layer. The travel direction, denoted Dir, was therefore treated as a process-state variable in the height prediction task.

### 2.2. Height Label Extraction and Sample Construction

After each layer was deposited, the wall geometry was scanned. The point cloud was denoised and trimmed in RevoScan5 (v5.5.4.1776) and then registered in CloudCompare (v2.13.2). The substrate plane was used as the reference plane; the deposition start point was set as the origin, and the deposition path was aligned with the X-axis. The registered point cloud was projected onto the X–Y plane, and the maximum Z value within each 0.5 mm path bin was extracted as the crown-height label to form a regular one-dimensional height sequence. This label represents model error relative to the adopted point-cloud processing workflow; repeated rescanning and alternative high-percentile labels should be evaluated in future work to quantify the measurement noise floor.

**Figure 3:**
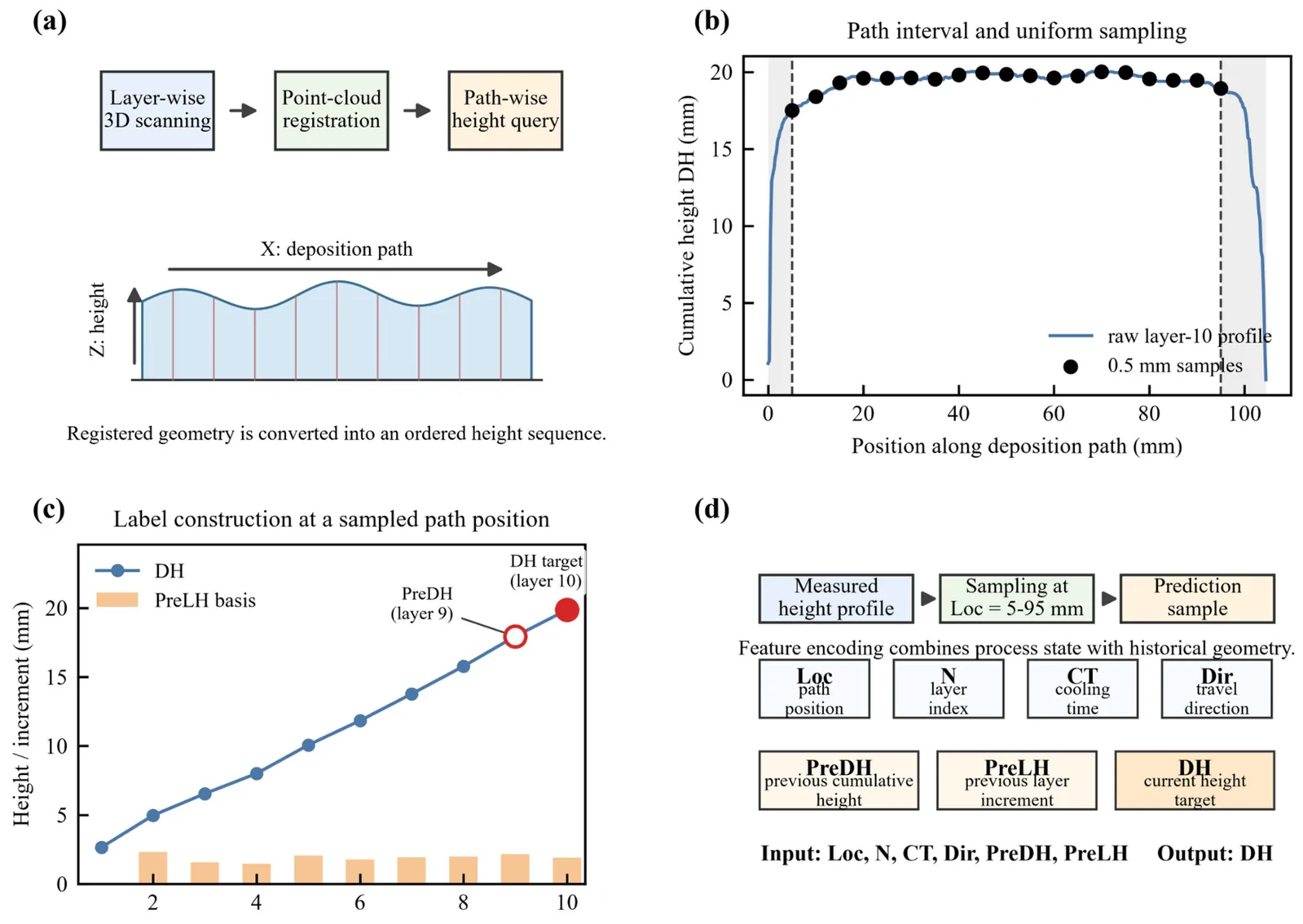
Height label extraction and path-wise sampling for deposited-height prediction: (**a**) Workflow from layer-wise 3D scanning to point-cloud registration and path-wise height query. (**b**) Extraction of the cumulative height sequence within the 5–95 mm modelling interval and uniform sampling at 0.5 mm spacing. (**c**) Construction of PreDH, PreLH and the current DH target at a sampled path position. (**d**) Encoding of the extracted height sequence into process-state, historical-geometry and target variables.

Figure 3 details how the height labels and model inputs were constructed from the measured wall geometry. Figure 3a summarizes the measurement workflow: each deposited layer was scanned; the point cloud was registered to a common coordinate system, and the height was queried along the deposition path. Figure 3b shows the extracted cumulative height profile and the modelling interval used in this work. The end regions were excluded from the modelling interval, and the 5–95 mm segment was sampled at 0.5 mm spacing to give a fixed-length path-wise sequence for each layer. Figure 3c illustrates the label definition at one sampled position. For a current layer, DH denotes the cumulative deposited height to be predicted. PreDH denotes the previous cumulative height at the same position, and PreLH denotes the previous layer height increment. Figure 3d then maps these extracted quantities, together with Loc, N, CT and Dir, into the multi-factor prediction sample used by the model.

The study focuses on single-bead multi-layer walls. Model training and evaluation used the 2, 3 and 4 min inter-layer cooling-time conditions. The inter-layer cooling times of 2, 3 and 4 min were selected based on preliminary experiments. In initial trials, cooling times below 2 min caused excessive heat accumulation and noticeable geometric distortion, while times above 4 min significantly reduced deposition efficiency without further substantial improvement in shape stability. The range of 2–4 min therefore represents a practically relevant window that balances thermal management and build productivity. The three discrete values were chosen to provide diverse thermal conditions for training the prediction model while keeping the experimental matrix manageable.

To reduce the influence of edge transients, the analyzed path interval was restricted to 5–95 mm; with a 0.5 mm sampling interval, each layer contained 181 sampled positions. CAFi-TCN does not use a sliding-window strategy: each deposited layer under one cooling-time condition is processed as one complete path-wise sequence with T = 181. Layers 1–8 were used for training, layer 9 for validation and layer 10 for testing. Therefore, the training set contained 24 sequences (4344 point-wise evaluations); the validation set contained 3 sequences (543 points), and the test set contained 3 sequences (543 points). These point-wise values describe spatial error distributions along the wall and should not be interpreted as independent replicate specimens.

**Figure 4:**
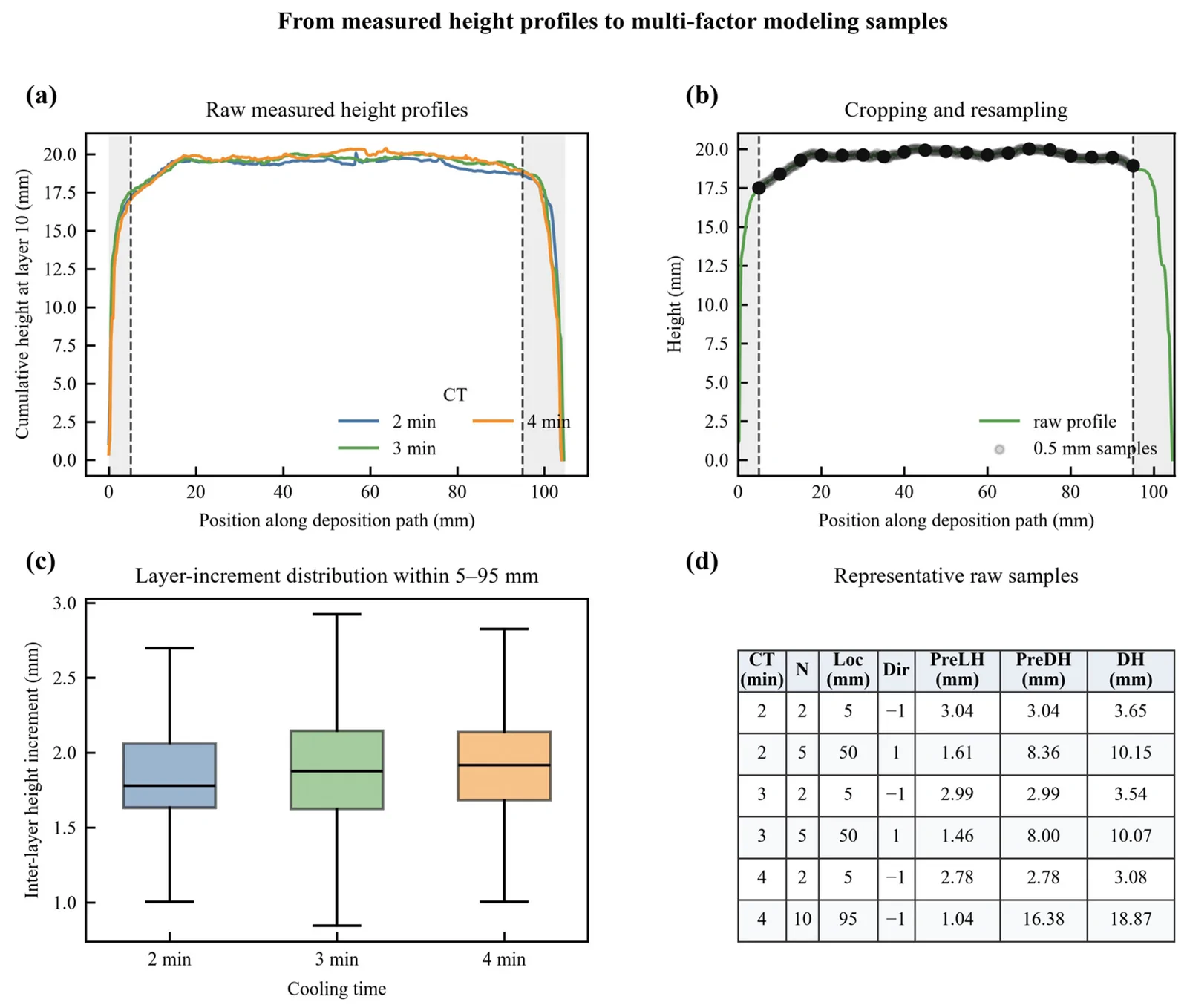
Construction of multi-factor modelling samples from measured height profiles: (**a**) Raw 10th-layer cumulative height profiles under different inter-layer cooling times, with the 5–95 mm modelling interval highlighted. (**b**) Cropping and 0.5 mm resampling of a representative measured height profile. (**c**) Distribution of inter-layer height increments within the modelling interval. (**d**) Six representative raw samples listing CT, N, Loc, Dir, PreLH, PreDH and DH.

Figure 4 expands the data-construction step from measured geometry to modelling samples. Figure 4a compares the raw 10th-layer cumulative height profiles extracted under 2, 3 and 4 min cooling-time conditions; the shaded end regions show why the modelling interval was restricted to 5–95 mm. Figure 4b illustrates the conversion of the raw measured profile into uniformly spaced path-wise samples at a 0.5 mm interval. Figure 4c summarizes the inter-layer height increments within the modelling interval. The distributions partly overlap across cooling-time conditions, indicating that CT contributes to the process state but does not alone determine the layer increment. Figure 4d lists six representative raw samples and reports the actual values of CT, N, Loc, Dir, PreLH, PreDH and target DH. The selected rows span the three cooling-time conditions, different layer numbers, the 5, 50 and 95 mm path positions, and both travel directions, thereby showing how the measured profiles were converted into model inputs and the corresponding prediction target.

**Figure 5:**
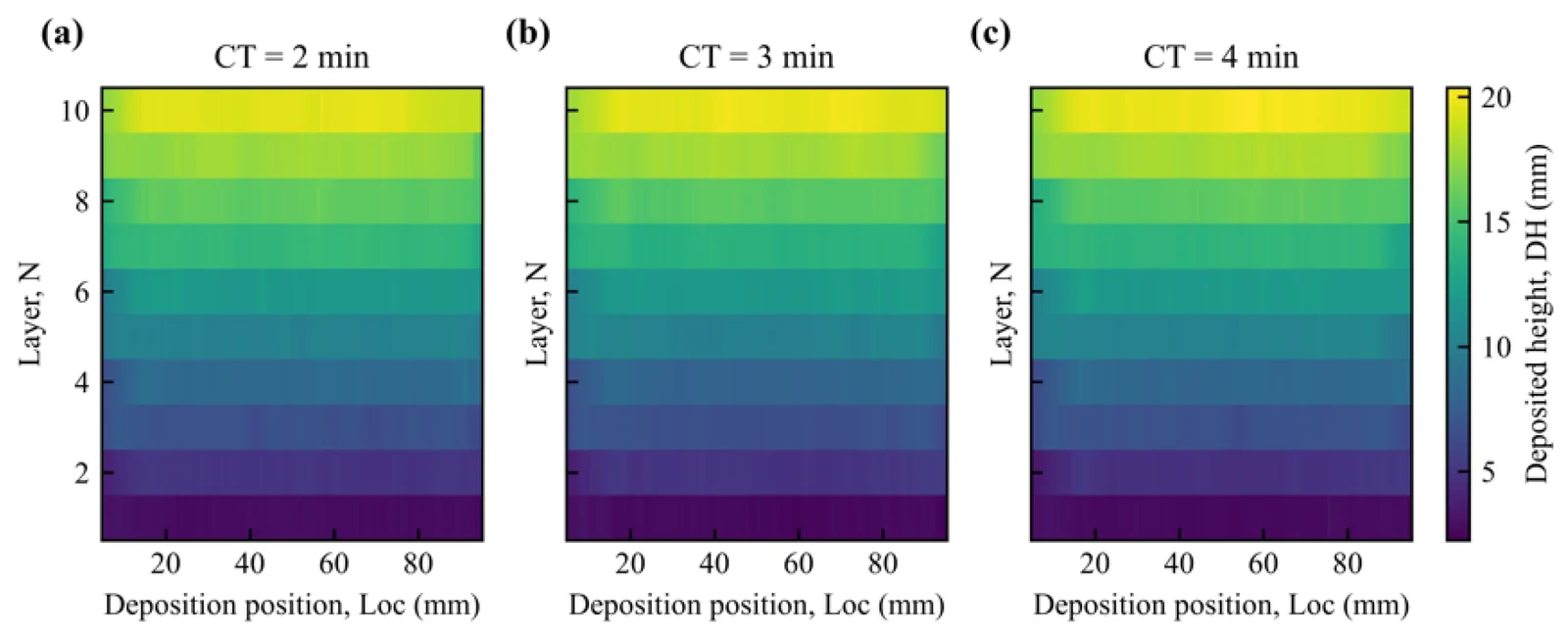
Layer-position height landscapes under different inter-layer cooling-time conditions: (**a**) 2 min. (**b**) 3 min. (**c**) 4 min.

As shown in Figure 5, the height maps show that the wall height rises with layer number, but not uniformly along the path. The middle region is comparatively stable, whereas the start and end regions show stronger local fluctuations. The 2, 3 and 4 min cases were used for model evaluation. The changes in layer-wise growth and path-wise fluctuation provide the basis for including CT as a process-state feature.

### 2.3. Model Architecture

**Figure 6:**
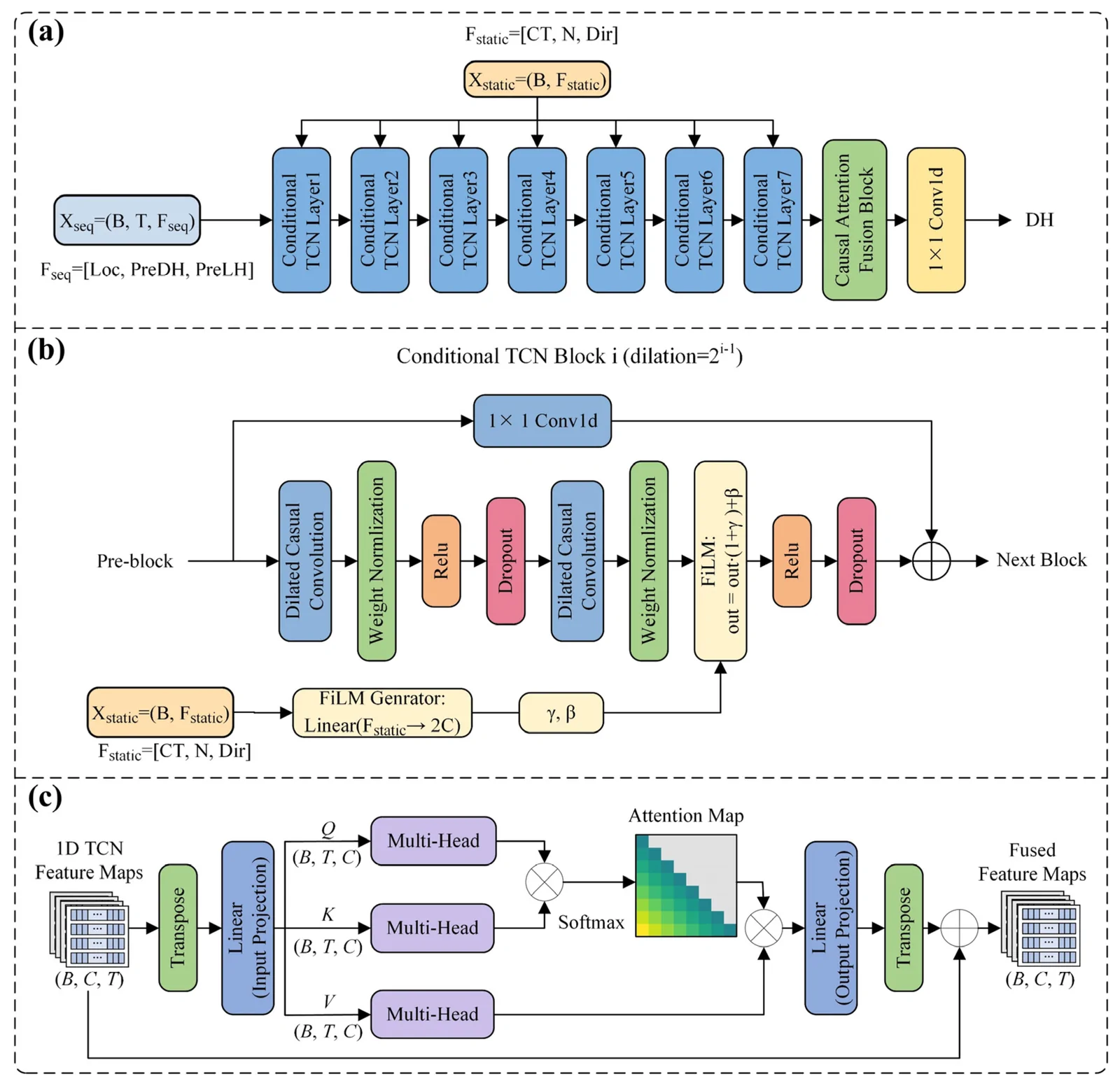
Architecture of CAFi-TCN for cumulative deposited-height prediction: (**a**) overall model architecture, (**b**) conditional TCN module architecture, and (**c**) causal attention architecture.

As illustrated in Figure 6, a CAFi-TCN model integrating linear modulation for process features and causal attention mechanism enhancement is proposed in this work. The proposed CAFi-TCN uses a TCN backbone. Loc, PreLH and PreDH are treated as an ordered spatial sequence along the deposition path, and N, CT and Dir are used as process-condition inputs. The sequence is not a time series in the conventional sense, but a path-ordered spatial sequence generated by the moving torch. In other words, the deposited profile records the process time history along the path: each later position is produced after the previous one and is influenced by preceding heat input, droplet spreading and start/end transients. This causal ordering provides the physical basis for using a temporal convolutional architecture. The FiLM module generates channel-wise modulation parameters from the process conditions to scale and shift intermediate features. The causal attention fusion block (CAFB) assigns weights to historical path positions under a causal constraint, so that the prediction at a current position is informed only by previous path states. The architecture addresses two questions: how process conditions change feature interpretation, and which historical locations should be emphasized for a given prediction position.

### 2.4. Baseline Models and Implementation Details

The compared models include MLP, LSTM, standard TCN, ridge regression, polynomial ridge regression, KNN, Random Forest, XGBoost and previous-height baselines. MLP, LSTM, TCN and CAFi-TCN were implemented in PyTorch (v2.5.1), while ridge regression, polynomial ridge regression, KNN and Random Forest used scikit-learn (v1.7.2) implementations. XGBoost used the XGBoost (3.1.3) package. All computations were performed using Python (v3.11.7) under WSL Ubuntu 22.04.5 on an NVIDIA GeForce RTX 4090 GPU. CAFi-TCN, MLP, LSTM and standard TCN were custom implementations developed by the authors using PyTorch. Ridge regression, polynomial ridge regression, KNN and Random Forest were implemented using scikit-learn, whereas XGBoost was implemented using XGBoost. NumPy and pandas were used for numerical operations and data handling, respectively. Neural-network models were trained with MSE loss and the AdamW optimizer [38]. Layers 1–8 were used to train the neural-network models; Layer 9 was used for validation and early stopping, and layer 10 was reserved for testing. Deterministic baselines were fitted using the available pre-test layers (layers 1–9) after their model forms were fixed, and all reported metrics were calculated on the same 10th-layer test set. For Random Forest and XGBoost, two target forms were evaluated: direct cumulative height (DH) and height increment (ΔDH = DH − PreDH). The direct variants are denoted RF-DH and XGBoost-DH, and the increment variants are denoted RF-Δ and XGBoost-Δ. We also added a deployable operator-style baseline, PreDH + mean(ΔDH_train), which reconstructs current height from the previous cumulative height and the mean training-set layer increment. A test-set mean-height oracle was reported only as a diagnostic reference because it uses target-set information. The archived CAFi-TCN configuration contains 289,665 trainable parameters. Its TensorBoard event record spans approximately 151 s from the first to the final logged event for the reported training run. The data split, model settings and evaluation metrics are listed in Table 3.

## 3. Results and Discussion

### 3.1. Height-Profile Tracking Under Different Cooling Times

**Figure 7:**
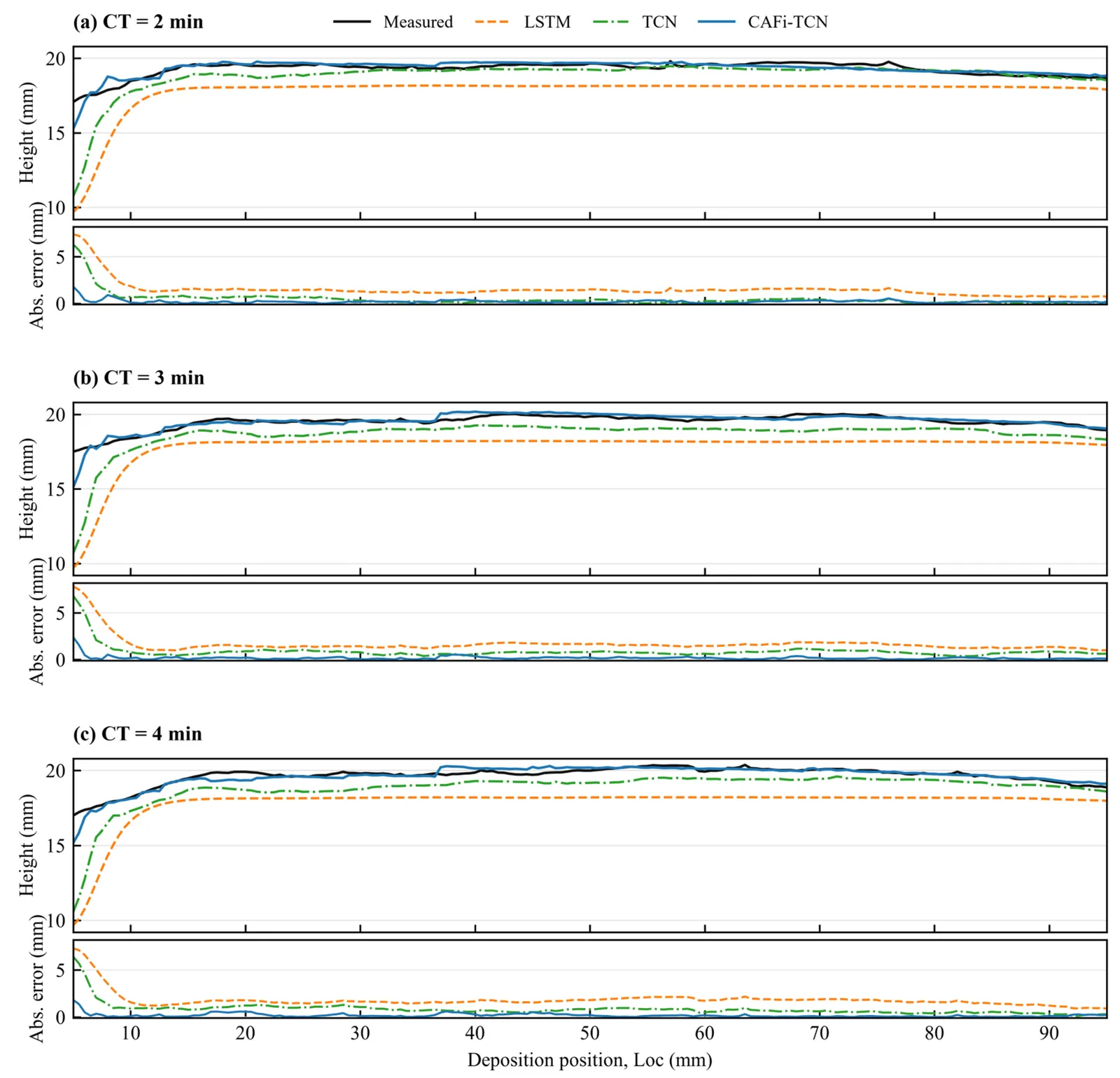
Measured and predicted 10th-layer height profiles under different inter-layer cooling times: (**a**) 2 min, (**b**) 3 min, and (**c**) 4 min.

Figure 7 compares the measured and predicted tenth-layer height profiles under inter-layer cooling times of 2, 3 and 4 min. Because most tenth-layer heights lie within a narrow 18–20 mm range, the main distinction between models appears in local deviations rather than in the absolute profile level. The measured profiles show a relatively stable middle section and more pronounced fluctuations near the path ends. LSTM and standard TCN follow the overall height level, but their deviations increase in the transient end regions and at local abrupt changes. CAFi-TCN matches the measured curves more closely under all three cooling-time conditions, especially in the stable middle region.

### 3.2. Model Comparison, Regional Errors and Feature Ablation

Table 4 summarizes the main model comparison and the tree-model target-form test in a single combined comparison. With MAE as the primary metric, CAFi-TCN achieved the lowest average absolute error (0.1836 mm) in the tenth-layer test set. Its MAE was lower than those of the standard TCN, polynomial ridge regression and MLP by 74.3%, 40.4% and 53.9%, respectively. The new PreDH + mean(ΔDH_train) baseline achieved MAE = 0.3190 mm, showing that a simple increment rule is a meaningful reference and is stronger than directly reusing the previous height. The RF-Δ result should be interpreted together with this result: RF-Δ achieved lower RMSE, higher R^2^ and a smaller maximum absolute error, whereas CAFi-TCN retained the best average-error performance.

A path-block bootstrap analysis was conducted to estimate uncertainty caused by spatial-segment variation along the test profiles. Using 5 mm path blocks and 2000 resamples, CAFi-TCN gave a bootstrap MAE of 0.183 mm (95% interval: 0.152–0.216 mm), while RF-Δ gave 0.216 mm (95% interval: 0.192–0.240 mm). The resulting intervals support the interpretation that CAFi-TCN is strongest in average-error control, whereas RF-Δ is a competitive increment-target baseline with lower tail risk.

**Figure 8:**
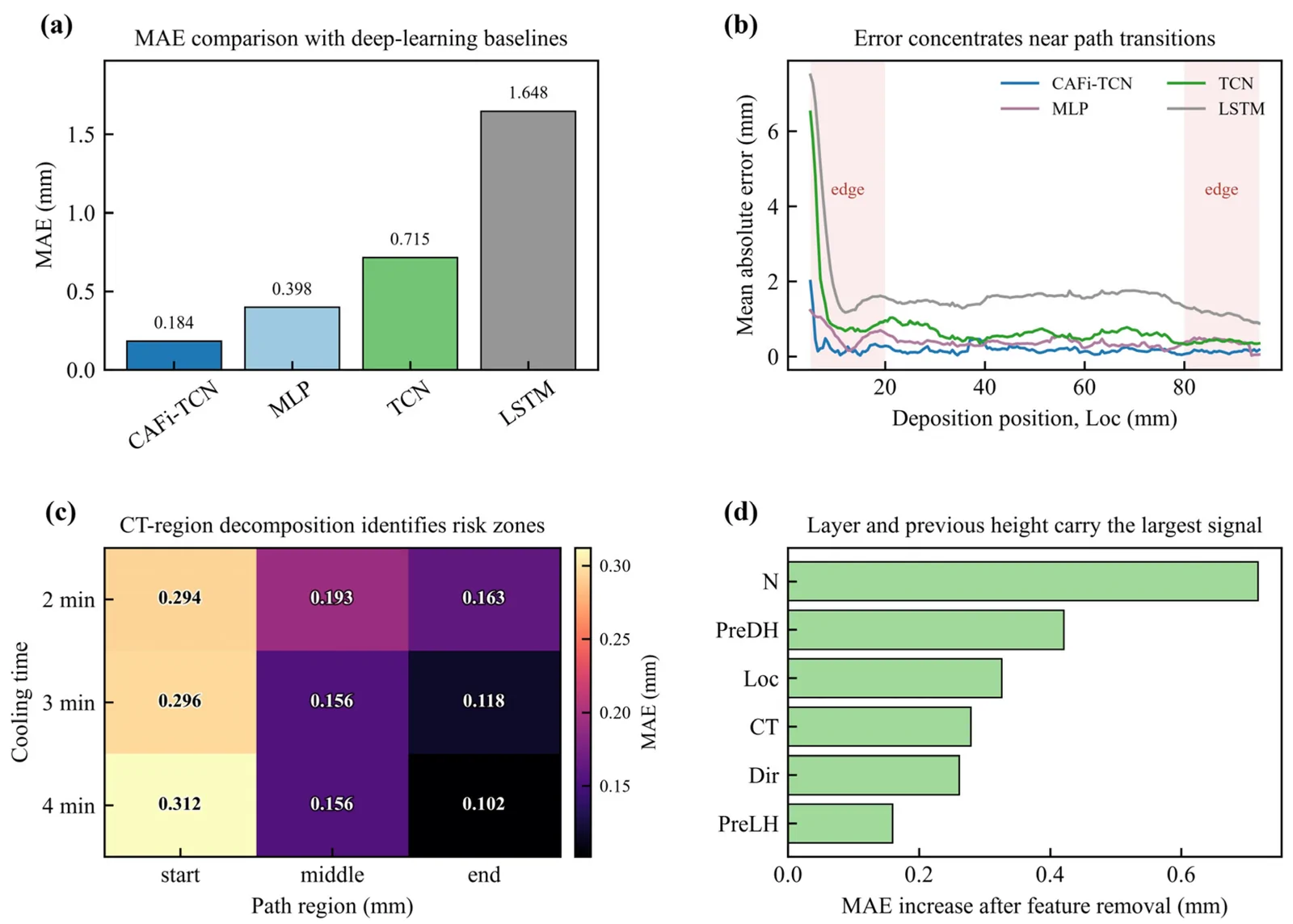
Model performance, path-wise error decomposition and feature ablation: (**a**) MAE comparison with deep-learning baselines. (**b**) Error concentrates near path transitions. (**c**) Regional error decomposition identifies risk zones: start region (5–20 mm), middle region (20–80 mm) and end region (80–95 mm). (**d**) Layer and previous height carry the largest signal.

Figure 8a compares the MAE values of the main learning models and shows the advantage of CAFi-TCN in average-error control. Figure 8b separates the prediction error by cooling time and path region, revealing that the start and end regions are more difficult than the middle region. Figure 8c evaluates the contribution of the FiLM and CAFB modules; removing either module increases the test error, which supports the use of condition modulation and causal attention fusion. Figure 8d further shows the feature-ablation results. The increases caused by removing N and PreDH indicate that layer accumulation and previous cumulative geometry carry the strongest predictive information.

### 3.3. Tree-Model Target Form, PreDH Audit and Rolling-Layer Validation

**Figure 9:**
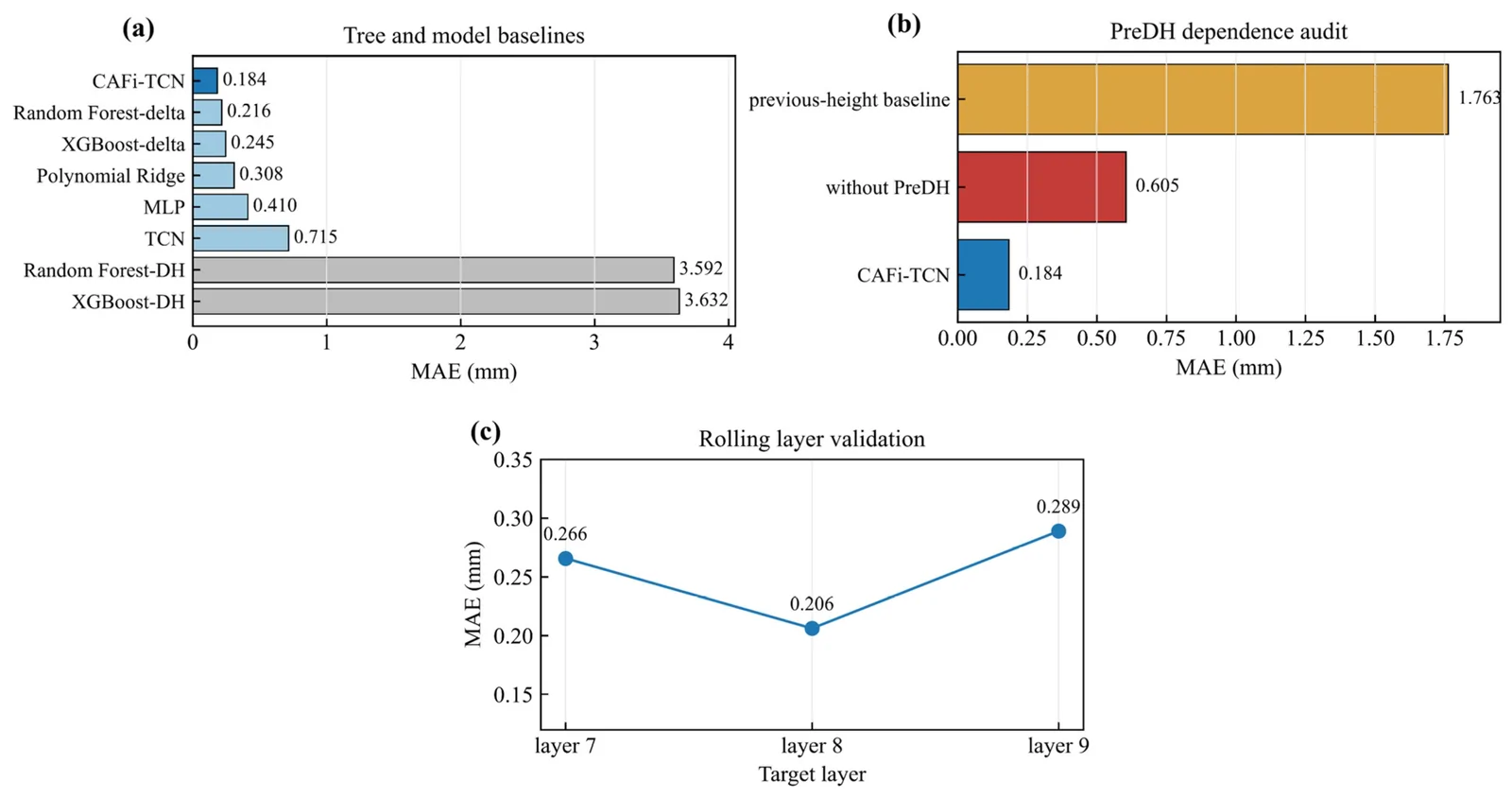
Tree-model baselines, PreDH dependency audit and rolling-layer validation: (**a**) tree-model baselines, (**b**) PreDH dependency audit, and (**c**) rolling-layer validation.

Tree-model baselines, simple increment baselines, a no-PreDH audit and rolling target-layer tests were used to check whether the results depend on one target form or one historical feature. Figure 9a shows that direct cumulative height prediction by XGBoost-DH and RF-DH produced MAEs of 3.6318 and 3.5922 mm, respectively. When the target was changed to the height increment ΔDH = DH − PreDH and PreDH was added back to reconstruct the current cumulative height, the MAEs decreased to 0.2449 mm for XGBoost-Δ and 0.2157 mm for RF-Δ. This comparison indicates that tree models have limited ability to extrapolate cumulative height outside the training-layer range but can fit layer-wise height increments effectively.

Because PreDH is an input feature, CAFi-TCN predictions were also evaluated after conversion to the height increment, ΔDH_pred = DH_pred − PreDH. On the same 543-point test set, the increment evaluation gave MAE = 0.1836 mm, RMSE = 0.2779 mm, R^2^ = 0.6300 and MaxAE = 2.3944 mm. The MAE, RMSE and MaxAE are identical to those of the DH evaluation because subtracting the same PreDH value from prediction and observation leaves the point-wise residual unchanged. The lower-increment-scale PA value (0.8959) reflects the smaller mean magnitude of ΔDH; PA is therefore retained only as an auxiliary normalized quantity.

Figure 9b presents the no-PreDH audit. Removing PreDH increased the CAFi-TCN MAE from 0.1836 mm to 0.6045 mm, confirming that previous cumulative height carries the strongest historical-geometry signal. The no-PreDH model nevertheless outperformed the previous-height baseline (MAE = 1.7634 mm), which shows that Loc, N, CT, Dir and PreLH still provide predictive information. Figure 9c reports MAEs of 0.2657, 0.2061 and 0.2889 mm for rolling target-layer validation on layers 7, 8 and 9, respectively. The model therefore does not rely only on the tenth-layer test set. Together, these results show that previous cumulative height is useful but not a stand-alone explanation for the model performance.

### 3.4. Error Distribution and Tail-Risk Analysis for End-Region Control

**Figure 10:**
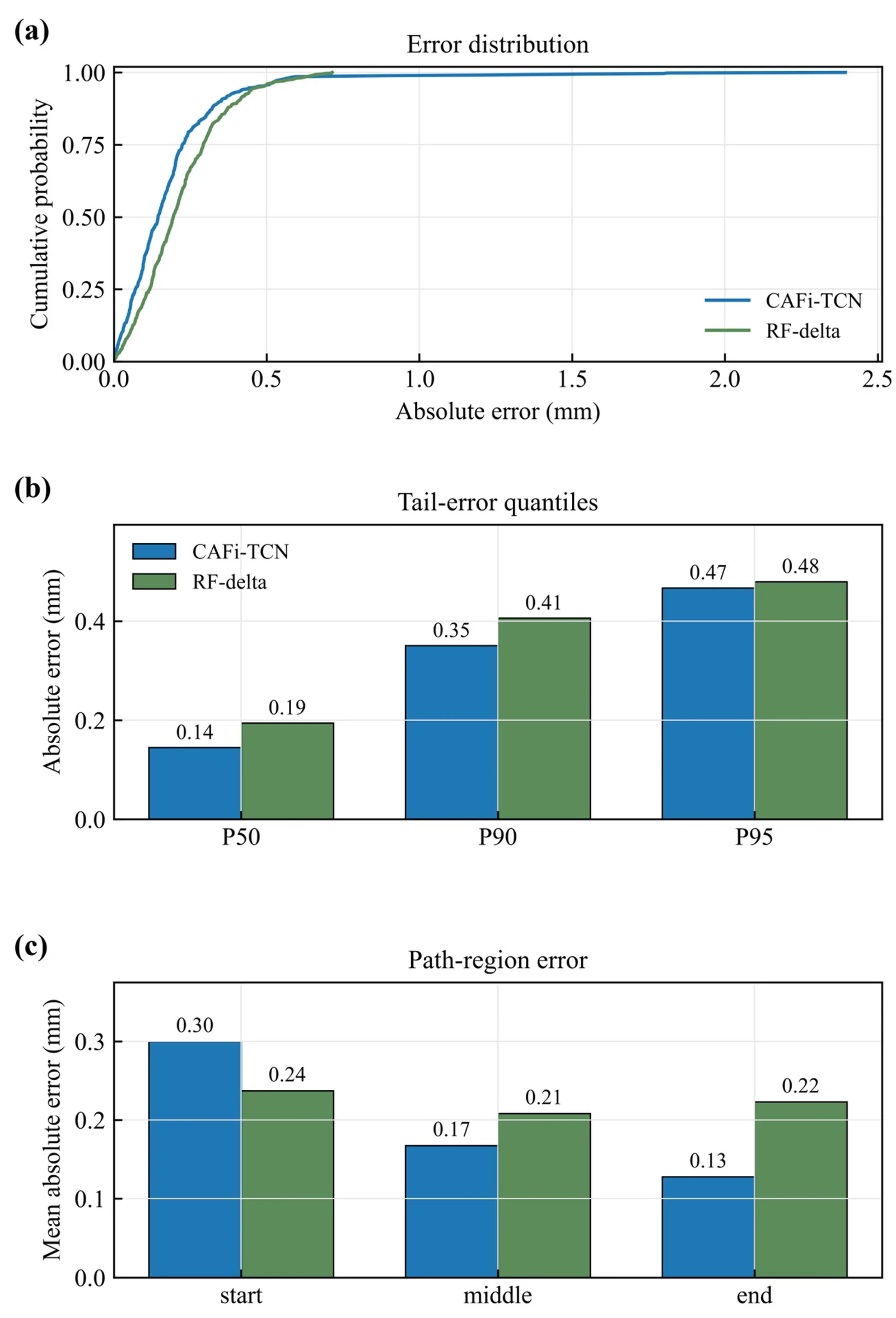
Error distribution, tail-error quantiles and path-region errors of CAFi-TCN and RF-Δ. (**a**) Error distribution. (**b**) Tail-error quantiles. (**c**) Path-region errors: start region (5–20 mm), middle region (20–80 mm) and end region (80–95 mm).

Although Table 3 and Table 4 provide global performance indicators, average errors can hide local large deviations. To compare the error patterns of CAFi-TCN and RF-Δ, the absolute-error distribution, tail-error quantiles and path-region errors were calculated for the tenth-layer test set, as shown in Figure 10. This analysis does not re-rank the models; it separates two engineering requirements: average-error control and extreme-error suppression.

The cumulative error distribution in Figure 10a rises more rapidly for CAFi-TCN in the low-error range, indicating that most positions have smaller prediction errors. Its longer tail, however, reveals a few large deviations. Figure 10b compares the 50th, 90th and 95th percentiles of absolute error, denoted P50, P90 and P95. These percentile errors give the thresholds below which 50%, 90% and 95% of the test samples fall. CAFi-TCN gives P50, P90 and P95 values of 0.14, 0.35 and 0.47 mm, whereas RF-Δ gives 0.19, 0.41 and 0.48 mm. The two models are therefore close over common error quantiles. Considering the maximum absolute error in Table 4, the advantage of RF-Δ mainly lies in suppressing extreme outliers rather than reducing every percentile error. Figure 10c shows that the average error in the start region is higher than that in the stable middle region, indicating that the start/end regions require special attention in path compensation and end-region process optimization.

The larger start/end-region errors are consistent with the transient nature of arc deposition. During arc ignition and termination, heat input, droplet transfer, molten-pool volume and wetting behaviour are less stable than in the middle of the path. At the start region, the molten pool develops from an initially colder surface; near the end region, local heat accumulation, torch residence effects and unstable pool spreading can change the final deposited geometry. These transients weaken the correspondence between historical geometry and current deposited height. End-region height prediction should therefore be treated as both a regression task and a process-specific problem related to transient heat accumulation and molten-pool stability.

### 3.5. Cooling-Time Response and Height Accumulation Trend

Inter-layer cooling time changes the time available for thermal relaxation before the next layer is deposited. In the present setup, CT was treated as a controllable process-state input rather than a direct temperature measurement. The CT response was examined by varying the CT input while deposition position, layer number, travel direction and historical height features were fixed.

**Figure 11:**
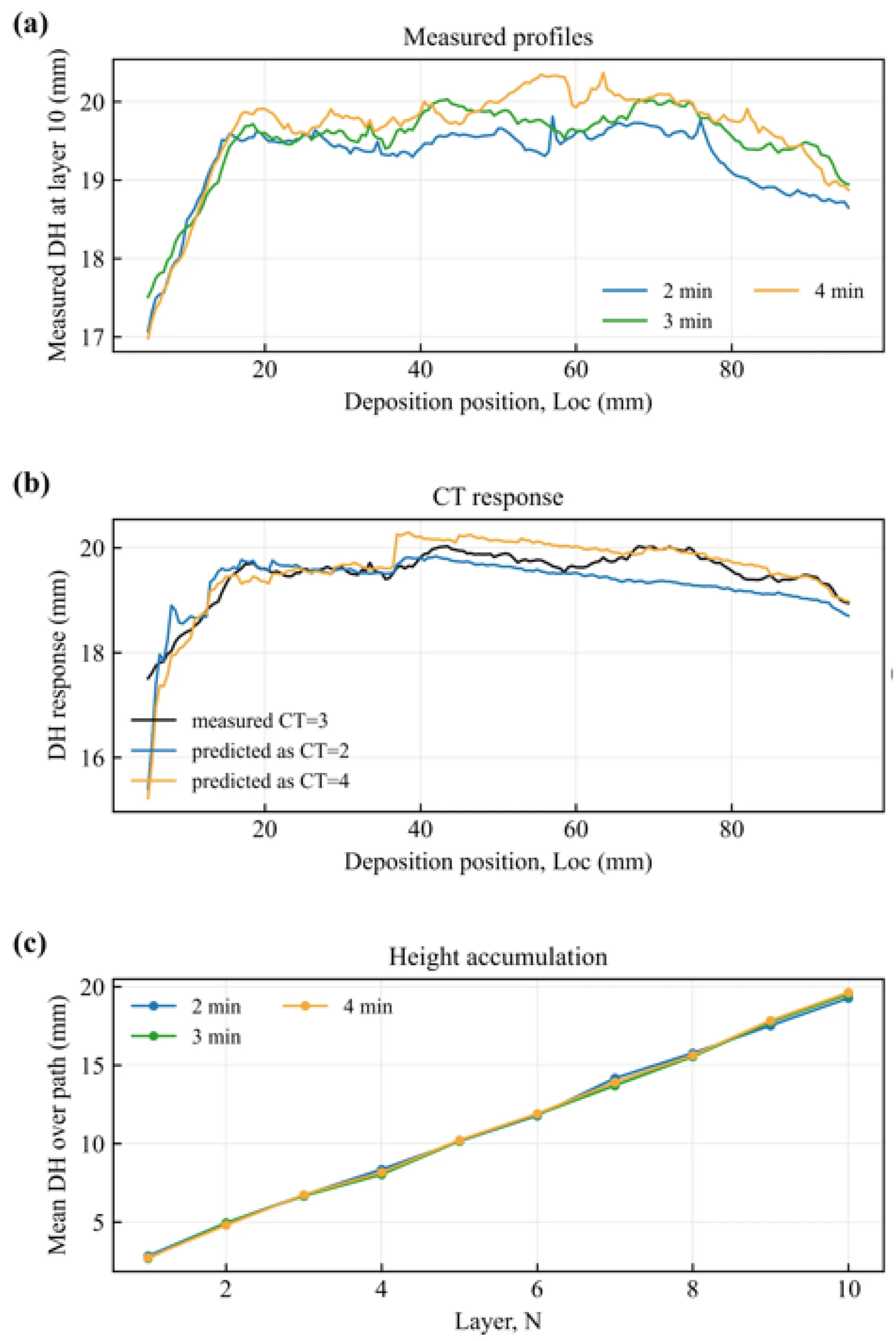
Influence of inter-layer cooling time on height distribution and model response. (**a**) Measured profiles. (**b**) CT response. (**c**) Height accumulation.

Figure 11a shows differences among the measured tenth-layer profiles under different cooling times, but the increment distributions overlap substantially. Across all analyzed layers, the mean height increments were 1.9272 ± 0.5178 mm, 1.9511 ± 0.5394 mm and 1.9640 ± 0.5566 mm for CT = 2, 3 and 4 min, respectively. For the tenth layer, the corresponding values were 1.7471 ± 0.4295 mm, 1.7574 ± 0.4862 mm and 1.7855 ± 0.4560 mm. These descriptive statistics show that CT is related to the process state within the investigated range, but the differences are modest relative to within-profile variability. Figure 11b therefore demonstrates model sensitivity to the CT input, whereas Figure 11c should be interpreted as a bounded height accumulation trend rather than proof that longer cooling always produces higher cumulative height.

A condition-level leave-one-CT-out analysis was additionally performed using the archived CAFi-TCN architecture. For each fold, layers 1–8 from two cooling-time conditions were used for training; layer 9 from those conditions was used for validation, and layer 10 from the held-out condition was used for testing. Holding out CT = 2, 3 and 4 min gave MAEs of 1.7972, 0.1845 and 3.7129 mm, respectively. The strongly condition-dependent result shows that the present three-condition dataset does not support a claim of reliable prediction at an unseen cooling-time setting. This analysis uses one wall per condition and is therefore a condition-level stress test, not an independent-build validation.

### 3.6. Engineering Implications and Limitations

This study has three engineering implications. First, the multi-factor input scheme links process state with historical geometry, matching the layer-wise accumulation behaviour of WAAM walls. Second, the path-wise error analysis identifies the end regions as critical locations for geometric control, which can guide local trajectory compensation and end-region process optimization. Third, the conditional response analysis shows how the model uses inter-layer cooling-time information, supporting trend assessment before parameter adjustment.

The conclusions remain bounded by the experimental scope. First, the study used single-bead multi-layer walls; multi-bead overlap, complex paths and other material systems were not evaluated. Second, the main test set is the tenth layer. The results demonstrate next-layer height prediction based on deposited-layer history, not unrestricted extrapolation to arbitrary components or process windows. Third, the available dataset contains only one wall per cooling condition, and the dense path sampling should not be interpreted as independent replicate specimens. The path-block bootstrap estimates spatial-segment uncertainty, but it does not quantify specimen-to-specimen variability. Fourth, the measurement noise floor of the 3D scanning and registration workflow was not established by repeated rescanning. Fifth, Figure 11 analyses height distribution and model response with respect to cooling time and should not be read as independent accuracy validation under an unseen operating condition. The leave-one-CT-out analysis further showed that performance was not consistent when an entire cooling-time condition was excluded from training; the model should therefore be used only within the represented cooling-time conditions until additional independently fabricated walls are available.

Inter-layer cooling time served as a controllable proxy for thermal condition because direct inter-layer temperature measurements were not available in the current setup. Under fixed ambient conditions, substrate geometry, wall geometry and deposition parameters, CT changes the thermal relaxation time before the next layer is deposited. Future work should incorporate infrared or thermocouple temperature sensing to test whether measuredinter-layerr temperature improves height-increment prediction.

## 4. Conclusions

This study addressed cumulative deposited-height prediction for single-bead multi-layer WAAM walls. CAFi-TCN combines process-condition modulation with causal attention-based historical information fusion. A multi-factor feature system was constructed from deposition position, layer number, cooling time, travel direction and historical geometry using a CMT-WAAM fabrication and layer-wise 3D scanning workflow.

Under the same data split and fixed random seed, CAFi-TCN achieved an MAE of 0.1836 mm in the tenth-layer test set. It obtained the lowest MAE among the evaluated direct-height models and outperformed LSTM, MLP, standard TCN, ridge regression, polynomial ridge regression, KNN and the previous-height baseline. The PreDH + mean (ΔDH_train) baseline reached MAE = 0.3190 mm, confirming that a simple average-increment rule is a strong baseline but still less accurate than CAFi-TCN in MAE. Compared with RF-Δ and XGBoost-Δ, CAFi-TCN retained the lowest MAE, whereas RF-Δ achieved better RMSE, R^2^ and maximum absolute error. The error distribution and path-block bootstrap analyses therefore support a balanced conclusion: CAFi-TCN controls average error more effectively, while RF-Δ suppresses extreme outliers more effectively.

The no-PreDH audit showed that removing the previous cumulative height increased the prediction error, but the no-PreDH model still outperformed the previous-height baseline. The model therefore did not simply copy the previous layer; it used position, layer number, cooling time, travel direction and previous layer increment jointly. Rolling target-layer validation also showed that the modelling route did not rely only on the tenth-layer test set, because historical geometry and process state remained informative across different target layers.

Overall, cumulative deposited-height prediction in WAAM should consider deposition position, inter-layer cooling time, travel direction, historical cumulative geometry and inter-layer height increment together. The combined evidence from model comparison, simple increment baselines, regional error analysis, tree-model increment baselines, PreDH audit, path-block bootstrap and rolling-layer validation supports bounded multi-factor process-state and historical-geometry modelling for deposited-height prediction and parameter-adjustment decision support.

## Figures and Tables

**Table 1:** Definitions of variables used for multi-factor height prediction.

Symbol	Meaning	Type	Role
Loc	Sampled position along the deposition path	Ordered spatial feature	Describes path-wise nonuniformity
N	Current layer number	Process-state feature	Represents heat accumulation and overall height background
CT	Inter-layer cooling time	Process-state feature	Represents the thermal state between layers
Dir	Travel direction	Process-state feature	Describes the spatial correspondence between start and end regions
PreLH	Height increment of the previous layer	Historical geometry feature	Reflects the local increment of the preceding layer
PreDH	Cumulative deposited height of the previous layer	Historical geometry feature	Reflects the local cumulative height background
DH	Cumulative deposited height of the current layer	Prediction target	Model output

**Table 2:** The chemical composition of the wire.

Element	C	Mn	Si	S	P	Cu	Ni	Cr	Mo	V
MG50-6	0.06–0.15	1.40–1.85	0.80–1.15	≤0.035	≤0.025	≤0.50	≤0.15	≤0.15	≤0.15	≤0.03

**Table 3:** Data split, model settings and evaluation metrics.

Item	Setting
Data range	CT = 2/3/4 min; N = 1–10; Loc = 5–95 mm; sampling interval = 0.5 mm
Training/validation/test	Neural models: N = 1–8/N = 9/N = 10; deterministic baselines fitted on pre-test layers N = 1–9 after model forms were fixed; all metrics evaluated on N = 10
Main model	CAFi-TCN: 7 TCN blocks, 64 channels, kernel size = 5, dropout = 0.2, learning rate = 3.32e-4, AdamW optimizer, batch size = 4, maximum epochs = 200
Deep-learning baselines	MLP, LSTM and standard TCN; all used the same train/validation/test layer split
Traditional baselines	Ridge, polynomial ridge, KNN, Random Forest, XGBoost and previous-height baseline; tree models were tested with DH and ΔDH targets; XGBoost: 500 trees, maximum depth = 3, learning rate = 0.03; Random Forest: 500 trees
Metrics	MAE, RMSE, PA, R^2^ and maximum absolute error
Sample units	Neural models: 24 training sequences/4344 point-wise evaluations, 3 validation sequences/543 points and 3 test sequences/543 points; T = 181 for each path-wise sequence
Normalization	Min–max normalization was fitted on the training set and then applied to validation and test data
Software implementation	Python scripts; PyTorch for MLP/LSTM/TCN/CAFi-TCN; scikit-learn for Ridge, polynomial Ridge, KNN and Random Forest; XGBoost package for XGBoost; RevoScan and CloudCompare for point-cloud processing
Training strategy	MSE loss, AdamW optimizer, maximum epochs = 200, early-stopping patience = 20, NVIDIA GeForce RTX 4090 under WSL Ubuntu 22.04.5
Uncertainty check	5 mm path-block bootstrap with 2000 resamples; this estimates spatial-segment uncertainty but not replicate-wall uncertainty
Model size and archived runtime	CAFi-TCN: 289,665 trainable parameters; archived TensorBoard event trace: approximately 151 s from first to final logged event for the reported run

**Table 4:** Combined prediction performance of the main model, conventional baselines and tree-model target forms on the 10th-layer test set.

Model	Target Form	Samples	MAE (mm)	RMSE (mm)	PA	R^2^	MaxAE (mm)
CAFi-TCN	DH	543	0.1836	0.2778	0.9906	0.7552	2.3941
RF-Δ	ΔDH + PreDH	543	0.2157	0.2570	0.9889	0.7906	0.7148
XGBoost-Δ	ΔDH + PreDH	543	0.2449	0.2940	0.9874	0.7259	0.7712
Polynomial ridge	DH	543	0.3083	0.3941	0.9840	0.5075	1.0750
PreDH + mean ΔDH_train	PreDH + mean training-set ΔDH	543	0.3190	0.4958	0.9836	0.2203	1.8959
Test-mean DH oracle	Test-set mean DH	543	0.3833	0.5615	0.9803	0.0000	2.4944
Ridge	DH	543	0.3850	0.4821	0.9800	0.2630	1.8139
MLP	DH	543	0.3980	0.5002	0.9796	0.2066	1.4648
TCN	DH	543	0.7149	1.0408	0.9633	−2.4352	6.8161
LSTM	DH	543	1.6478	1.8909	0.9154	−10.3396	7.8184
Previous-height baseline	PreDH	543	1.7634	1.8216	0.9095	−9.5230	3.1000
RF-DH	DH	543	3.5922	3.6064	0.8155	−40.2480	4.4191
XGBoost-DH	DH	543	3.6318	3.6527	0.8135	−41.3145	4.4523
KNN	DH	543	3.7838	3.8013	0.8059	−44.8260	4.4368

## Data Availability

The original contributions presented in this study are included in the article. Further inquiries can be directed to the corresponding authors.

## References

[1] Williams S.W., Martina F., Addison A.C., Ding J., Pardal G., Colegrove P. (2016). Wire + arc additive manufacturing. Mater. Sci. Technol..

[2] DebRoy T., Wei H.L., Zuback J.S., Mukherjee T., Elmer J.W., Milewski J.O., Beese A.M., Wilson-Heid A., De A., Zhang W. (2018). Additive manufacturing of metallic components—Process, structure and properties. Prog. Mater. Sci..

[3] Herzog D., Seyda V., Wycisk E., Emmelmann C. (2016). Additive manufacturing of metals. Acta Mater..

[4] Wu B., Pan Z., Ding D., Cuiuri D., Li H., Xu J., Norrish J. (2018). A review of the wire arc additive manufacturing of metals: Properties, defects and quality improvement. J. Manuf. Process..

[5] Xia C., Pan Z., Li Y., Polden J., Zhang D., Chen S. (2020). A review on wire arc additive manufacturing: Monitoring, control and a framework of automated system. J. Manuf. Syst..

[6] Li Y., Su C., Zhu J. (2022). Comprehensive review of wire arc additive manufacturing: Hardware system, physical process, monitoring, property characterization, application and future prospects. Results Eng..

[7] He F., Yuan L., Mu H., Ros M., Ding D., Pan Z., Li H. (2023). Research and application of artificial intelligence techniques for wire arc additive manufacturing: A state-of-the-art review. Robot. Comput.-Integr. Manuf..

[8] Chen Z., Chen Z., Yuan L., Pan Z., Zhu H., Ma N., Ding D., Li H. (2025). A comprehensive review and future perspectives of simulation approaches in wire arc additive manufacturing (WAAM). Int. J. Extrem. Manuf..

[9] Ding D., Pan Z., Cuiuri D., Li H. (2015). A practical path planning methodology for wire and arc additive manufacturing of thin-walled structures. Robot. Comput.-Integr. Manuf..

[10] Si J., Luo L., Liu C., Lu J. (2025). Arc length monitoring and closed-loop quality control for GTA-WAAM. Virtual Phys. Prototyp..

[11] Chen D., Chen H., Zhang G., Xiong J. (2025). Towards automatic control of process stability for thick-wall parts in arc-directed energy deposition based on arc voltage sensing. J. Manuf. Process..

[12] Xiong J., Yin Z., Zhang W. (2016). Closed-loop control of variable layer width for thin-walled parts in wire and arc additive manufacturing. J. Mater. Process. Technol..

[13] Zhang L., Sun M., Wang H., Wang J., Bian W., Dai X. (2024). A layer superposition strategy based on a theoretical model for wire and arc additive manufacturing of multi-layer single-bead components. CIRP J. Manuf. Sci. Technol..

[14] Zhang H., Bai X., Dong H., Zhang H. (2024). Modelling and prediction of process parameters with low energy consumption in wire arc additive manufacturing based on machine learning. Metals.

[15] So M.S., Mahdi M.M., Kim D.B., Shin J.H. (2024). Prediction of metal additively manufactured bead geometry using deep neural network. Sensors.

[16] Gihr M., Rashid A., Melkote S.N. (2024). Bead geometry prediction and optimization for corner structures in directed energy deposition using machine learning. Addit. Manuf..

[17] Kitagawa H., Malek T., Kono D., Denkena B. (2026). 3D geometry prediction for wall deposition using transfer learning in wire arc additive manufacturing. CIRP J. Manuf. Sci. Technol..

[18] Wang Y., Zhang C., Lu J., Bai L., Zhao Z., Han J. (2020). Weld reinforcement analysis based on long-term prediction of molten pool image in additive manufacturing. IEEE Access.

[19] Dong K., Wu Q., Qin X., Hu Z., Hua L. (2024). In-situ optical monitoring and analysis of weld pool based on machine vision for wire and arc additive manufacturing. Int. J. Adv. Manuf. Technol..

[20] Yu M., Zhu L., Ning J., Yang Z., Jiang Z., Xu L., Wang Y., Meng G., Huang Y. (2024). Machine vision and novel attention mechanism TCN for enhanced prediction of future deposition height in directed energy deposition. Mech. Syst. Signal Process..

[21] Wang Y., Hu K., Li W., Wang L. (2023). Prediction of melt pool width and layer height for laser directed energy deposition enabled by physics-driven temporal convolutional network. J. Manuf. Syst..

[22] Yu M., Zhu L., Yang Z., Lu X., Ning J., Chang B. (2024). A novel data-driven framework for enhancing the consistency of deposition contours and mechanical properties in metal additive manufacturing. Comput. Ind..

[23] Pang B., Zeng Z., Zhou N., Wu Y., Chen L., Zhang X., Shen J., Ji Q., Yin M. (2026). Prediction of 3D as-deposited layer geometry with sequential SDF-based spatio-temporal networks in wire arc additive manufacturing. Addit. Manuf. Front..

[24] Liu M., Bai X., Xi S., Dong H., Li R., Zhang H., Zhou X. (2024). Detection and quantitative evaluation of surface defects in wire and arc additive manufacturing based on 3D point cloud. Virtual Phys. Prototyp..

[25] Mattera G., Polden J., Norrish J. (2024). Monitoring the gas metal arc additive manufacturing process using unsupervised machine learning. Weld. World.

[26] Franke J., Heinrich F., Reisch R.T. (2025). Vision based process monitoring in wire arc additive manufacturing (WAAM). J. Intell. Manuf..

[27] Gao F., Li C., Ji D., Hua J., Lin J. (2025). Spectral characterization method for wire-arc additive manufacturing monitoring with broadband AE signals. Meas. Sci. Technol..

[28] Tapia Cabrera J.E., Groschupp F., Riegger F., Zaeh M.F. (2026). AI-based monitoring system for real-time defect detection in wire arc additive manufacturing. J. Adv. Join. Process..

[29] Afzal M.A., Mukherjee T. (2026). A review on in-situ monitoring in wire arc additive manufacturing: Technologies, applications, challenges, and needs. Machines.

[30] Kishor G., Mugada K.K., Mahto R.P. (2025). Sensor-integrated data acquisition and machine learning implementation for process control and defect detection in wire arc-based metal additive manufacturing. Precis. Eng..

[31] Arthur N.J.R.S., Liu L., Oliveira J.P. (2025). Design and development of open-source, multi-purpose wire arc additive manufacturing machine. Int. J. Adv. Manuf. Technol..

[32] Li Y., Li K., Chen C., Zhou X., Zeng Z., Li K. (2021). Modeling temporal patterns with dilated convolutions for time-series forecasting. ACM Trans. Knowl. Discov. Data.

[33] Hochreiter S., Schmidhuber J. (1997). Long short-term memory. Neural Comput..

[34] Niu Z., Zhong G., Yu H. (2021). A review on the attention mechanism of deep learning. Neurocomputing.

[35] Garcia D., Perez D., Papapetrou P., Diaz I., Cuadrado A.A., Enguita J.M., Dominguez M. (2025). Conditioned fully convolutional denoising autoencoder for multi-target NILM. Neural Comput. Appl..

[36] Mattera G., Nele L. (2025). Machine learning approaches for real-time process anomaly detection in wire arc additive manufacturing. Int. J. Adv. Manuf. Technol..

[37] Song H., Li C., Fu Y., Li R., Zhang H., Wang G. (2023). A two-stage unsupervised approach for surface anomaly detection in wire and arc additive manufacturing. Comput. Ind..

[38] Barakat A., Bianchi P. (2021). Convergence and dynamical behavior of the ADAM algorithm for nonconvex stochastic optimization. SIAM J. Optim..

